# Supplementary material for: Development and Validation of a Bedside Scale for Assessing Upper Limb Function Following Stroke: A Methodological Study
Source: F1000Res. 2024 Jun 3;13:565. [Version 1] doi: 10.12688/f1000research.149935.1 (PMC11803442; doi:10.12688/f1000research.149935.1)
Supplement: Peer review history

# Reviewer report: Reviewer response for version 1

**DOI:** 10.5256/f1000research.164449.r288418

**Published:** 2025-01-02

**Authors:** Anuprita Kanitkar

**Affiliations:** 1 Department of Applied Health Sciences, University of Manitoba, Winnipeg, MB, Canada

This study introduces the Bedside Upper Limb Evaluation Tool (BUFET) as a method for assessing upper limb (UL) function in stroke survivors. The BUFET focuses on common gross movements such as everyday functional gestures, (but not object manipulation tasks of daily living) providing a practical, cost-effective alternative to more complex and expensive assessments. The psychometric properties were. assessed by comparing it to the Wolf Motor Function Test (WMFT), demonstrating strong concurrent validity (r = 0.937, p < 0.001) and excellent internal consistency (Cronbach’s alpha = 0.948). By evaluating movements like hand gestures, finger tapping, and wrist movements, BUFET helps identify basic motor impairments and suggests possible locations of brain lesions. Its quick administration and comprehensive assessment of both proximal and distal motor control make it suitable for use in clinical, research, and home settings. The study shows the BUFET’s potential to aid in rehabilitation assessments in stroke care, though further research is needed to explore its reliability and prognostic value. 

 The study is well-executed and well-written. The study design is robust. the results for the concurrent validity and internal consistency are presented clearly. The BUFET is a good assessment tool to evaluate the mobility in the upper extremity after a recent stroke. as a bedside assessment tool, it requires minimal equipment and can be performed in minutes, which speaks for its usability, especially considering it showed excellent concurrent validity and internal consistency. 

 I approve this study as scientifically valid in its current form. The study is well-planned and implemented. I think it needs a thorough grammar check once.

Is the work clearly and accurately presented and does it cite the current literature?

Yes

If applicable, is the statistical analysis and its interpretation appropriate?

I cannot comment. A qualified statistician is required.

Are all the source data underlying the results available to ensure full reproducibility?

Yes

Is the study design appropriate and is the work technically sound?

Yes

Are the conclusions drawn adequately supported by the results?

Yes

Are sufficient details of methods and analysis provided to allow replication by others?

Yes

Reviewer Expertise:

NA

I confirm that I have read this submission and believe that I have an appropriate level of expertise to confirm that it is of an acceptable scientific standard.

# Reviewer report: Reviewer response for version 1

**DOI:** 10.5256/f1000research.164449.r342904

**Published:** 2024-12-13

**Authors:** Dr. Sharmila Dudhani

**Affiliations:** 1 SDM College of Physiotherapy, SDM University, Dharwad, India

The article introduces a novel and user-friendly approach that holds significant promise for practical application. Its methodology and findings are both compelling and relevant to the field.

 I suggest some clarifications as follows: 

 1. Will this scale classify the involvement of hand as mild, moderate and severe affection?

 2. Please do mention about the instruction to the patient.

 3.Are the items culturally appropriate and sensitive for the intended population?

Is the work clearly and accurately presented and does it cite the current literature?

Yes

If applicable, is the statistical analysis and its interpretation appropriate?

Yes

Are all the source data underlying the results available to ensure full reproducibility?

Yes

Is the study design appropriate and is the work technically sound?

Yes

Are the conclusions drawn adequately supported by the results?

Yes

Are sufficient details of methods and analysis provided to allow replication by others?

Yes

Reviewer Expertise:

qualitative health research in physiotherapy

I confirm that I have read this submission and believe that I have an appropriate level of expertise to confirm that it is of an acceptable scientific standard.

# Response

**Published:** 2024-12-22

**Authors:** vijayakumar palaniswamy

**Affiliations:** Physiotherapy, Kasturba Hospital Manipal, Mangalore, Karnataka, India

Dear Dr., Dudhani,

 Thank you for your thoughtful review and constructive feedback on our manuscript. Below are our responses to your clarifications:

**1. Will this scale classify the involvement of the hand as mild, moderate, and severe affection**?

**Author’s Response**:

 While the current scale does not classify hand involvement into mild, moderate, and severe categories, we acknowledge its potential value. Our primary aim was to provide a functional assessment tool. However, future versions of the scale may explore incorporating severity classifications.

**2. Please do mention the instructions to the patient.**

**Author’s Response**:

 Thank you for your feedback regarding the patient instructions for the BUFET. We believe the instructions within the tool are already clear and do not require any changes. However, in response to your suggestion, we have added an overview of the scale's intended purpose and instructions for patients. This overview explains the goals of the BUFET, the types of tasks involved, and how patients should approach the evaluation.

 The added overview ensures patients understand the scale's purpose and what is expected during its use. This information has been incorporated into the appendix of the manuscript and aligns with your recommendations to enhance the clarity and accessibility of the BUFET.

 .

**3. Are the items culturally appropriate and sensitive for the intended population?**

**Author’s Response**:

 Thank you for your thoughtful feedback regarding the cultural appropriateness of the BUFET. While the original development of the scale did not explicitly aim to test cultural sensitivity, we believe the current version is inherently culturally appropriate and sensitive for the intended population, based on the following considerations:

**Item Design and Universality**:

 The tasks included in the BUFET, such as salute, hand waving, and pointing the index finger, represent universally understood and culturally neutral gestures or actions. These tasks are commonly performed in daily life and do not require a specific cultural context for interpretation.

**Expert Validation:**

 During the development phase, the scale was reviewed by a diverse panel of rehabilitation experts, including neurologists and physiotherapists with extensive experience working within the intended population. This process ensured that all items were appropriate and relevant for the local cultural context.

 We hope these responses address your concerns. Thank you for your valuable insights,

 Sincerely,

 Dr Vijayakumar Palaniswamy
